# The Postharvest Quality of Fresh Sweet Cherries and Strawberries with an Active Packaging System

**DOI:** 10.3390/foods8080335

**Published:** 2019-08-09

**Authors:** Valentina Chiabrando, Luigi Garavaglia, Giovanna Giacalone

**Affiliations:** 1 Department of Agricultural, Forestry and Food Sciences, University of Torino, Largo Braccini 2, 10095 Grugliasco, Italy; 2 Ilip s.r.l., Via Castelfranco, 52, 40053 Valsamoggia (BO), Italy

**Keywords:** active pad, equilibrium modified atmosphere packaging, Life^+^ system, container, shelf life, storage

## Abstract

This study assessed the effect of the recently-introduced Life^+^ (ILIP, Valsamoggia, Italy) active packaging system on the postharvest quality of sweet cherries and strawberries. This system uses Equilibrium Modified Atmosphere Packaging (EMAP) to achieve specific intra-package conditions with three synergistic elements: an unvented and anti-mist heat sealable container, an active (naturally-antimicrobial) pad, and a heat-sealed, laser micro-perforated film of a specified gas permeability. Post-packaging quality parameters were monitored for 10 (strawberries) and 15 days (cherries): headspace gas concentration, weight loss, titratable acidity, total soluble solids, pH, disease incidence, and sensory quality. Results showed that use of the Life^+^ system delayed postharvest senescence by maintaining fruit color, acidity, and vitamin C content, and decreasing fruit weight loss and decay. The use of EMAP in sweet cherry resulted in enhanced sensory qualities compared to traditional perforated containers. The results suggest that the Life^+^ system leads to better sensory properties and improved shelf-life for strawberries and sweet cherries.

## 1. Introduction

The physiological characteristics of sweet cherries and strawberries make both fruits highly perishable and more susceptible to surface fungal spoilage during cold storage in comparison to other crops [1]. Not surprisingly, the postharvest storage periods of these fruits are limited by factors such as water loss, softening, color loss, stem browning, and surface pitting [2], as well as by diseases caused by common plant pathogens, in particular *Monilinia laxa, Botrytis cinerea, Alternaria alternate, Penicillium expansum, Rhizopus stolonifera*, and *Cladosporium* spp. [3]. Recently-created European legislation [4] restricts the use of synthetic fungicides to control postharvest diseases, which has shifted the focus of research to the development of innovative and sustainable strategies to preserve fruit and vegetable quality through non-chemical means.

To extend and preserve the shelf-life of strawberries and cherries, several technologies have been tested, such as treatment with edible coatings, calcium dipping, UV radiation, ultrasound, hot water, and modified atmosphere packaging [2,5,6,7,8]. Among these, MAP (Modified Atmosphere Packaging) and EMAP (Equilibrium Modified Atmosphere Packaging) are the most commonly used methods to prolong the postharvest life of fresh produce [9]. EMAP is MAP technology augmented with a permeable film that allows the O_2_ and CO_2_ transmitted through the film to be balanced with the respiration rate of the product or fruit [10,11].

Antimicrobials embedded in the film, or a label release active compound, is a new form of active packaging. This packaging interacts with the packaged food or the package headspace in order to reduce, retard, or even inhibit the growth of spoilage and pathogenic microorganisms. This antimicrobial active packaging can take several forms, including direct incorporation of the active compound into the polymer matrix, coating it onto the packaging surface, or immobilizing it in sachets or pads. Among the antimicrobial agents used, compounds of natural origin, such as several components of plant essential oils (EO) and food aromas, are preferred [12,13]. The antimicrobial activity of EOs is attributed to their major phenolic compounds (e.g., thymol, carvacrol, and eugenol), present in concentrations as high as 85% [14]. Medeiros et al. [15] developed antifungal sachets incorporated with oregano and lemongrass essential oils as part of a packaging system used for mangoes and achieved a reduction of fungal growth during storage. Seo et al. [16] developed an antimicrobial sachet incorporated with encapsulated AITC (allyl isothiocyanate) for treating spinach leaves, resulting in a reduction of microorganisms. Absorbent pads incorporated with copper nanoparticles showed positive effects on melon, kiwi, and pineapple preservation [17].

ILIP S.r.l. (Valsamoggia, Bazzano (BO), Italy) is a firm that has expanded the concept of EMAP with its newly-introduced Life^+^ packaging system. This system is based on three different pack components: an unvented and anti-mist heat sealable container, an active (antimicrobial) pad, and a heat-sealed laser-perforated film. This is the only system that combines a heat-sealed film with the use of an antimicrobial pad inside a food container. The makers of Life^+^ technology claim that their packaging increases food quality and extends shelf-life compared to traditional packaging, by reducing metabolism and inhibiting microorganisms.

To this end, our research evaluated the effects of the Life^+^ technology (EMAP + antimicrobial pad) on the quality of strawberries and sweet cherries during storage.

## 2. Materials and Methods

### 2.1. Plant Material

The sweet cherry (cv. Ferrovia) and strawberry (cv. Marmolada) fruit for this study were hand-picked from commercial orchards located in Vignola (Modena, Italy) and Peveragno (Cuneo, Italy), respectively. Each fruit type was selected for its uniformity of ripeness and size, as well as the absence of physical injuries and microbial infections. The samples were randomly distributed into PET containers with approximately 300 g of fruit in each. In total, there were 90 containers for cherries and 60 for strawberries. The first half of the fruit was placed in Life^+^ PET containers (16 cm × 9 cm × 9 cm) containing an antimicrobial pad on the bottom. The containers were then sealed with laser-perforate film Topaz 421 (21 µm of thickness with 25 holes of 70 µ per linear meter and an OTR of 2397.18 cc/m^2^/d) (Plastopil–Israel). The other half of the fruit was placed in traditional perforated containers with hinged lids and pressure closures (16 cm × 10 cm × 8 cm) (study control group). The traditional containers included 20 macro-holes, 4 circular holes (8 mm) at the bottom of the tray, and 16 holes at the top lid (4 circular holes of 8 mm and 12 rectangular holes of 5 mm × 15 mm).

The cherry and strawberry samples were stored for 15 and 10 days, respectively, in a cold room at 6 °C and 90%–95% R.H.

### 2.2. Gas Monitoring

The oxygen (O_2_) and carbon dioxide (CO_2_) concentrations in the headspace of the packaged samples were monitored daily using a portable CheckPoint II gas analyzer (Dansensor, Segrate, Italy). A needle attached to the gas analyzer was inserted through a rubber septum into the package. Three replicates per treatment (total = 6) were performed each day throughout the duration of storage. Results were expressed as the average O_2_ and CO_2_ kilopascals for the three replicates.

### 2.3. Weight Loss

Weight loss was determined by weighing the fruit at 0, 5, and 15 days for cherries and at 0, 5, and 10 days for strawberries. Results were expressed as the percentage of weight lost relative to the initial weight, according to Equation (1):

WL (%) = 100 × W_0_ − W_t_/W_0_ (1)

where WL is the percentage of weight lost, W_0_ is the initial weight of the fresh fruit sample, and W_t_ is the weight of the fruit sample at time t.

### 2.4. Color Measurements

Color analysis was performed after 0, 5, 10, and 15 days for cherries and after 0, 5, and 10 days for strawberries. The characteristics of color [L*, a*, b* and chroma (C*)] were measured at two points along the equatorial section of the fruit skin with a chroma meter, Illuminant C (Minolta, model CR-400, Tokyo, Japan). Chromatic analyses were conducted in accordance with the Commission Internationale de l’Eclairage (CIE) system. The C * value, calculated with the formula:

C* = (a*^2^  +  b*^2^)^1/2^ (2)

was used to represent the color saturation [18]. For each fruit type, color characteristic measurements resulted from the average ± SD of 30 measurements of each treatment taken at each time.

### 2.5. Quality Measurements

The total soluble solids (%) and titratable acidity (meq L^−1^) were measured on day 0, 5, 10, and 15 for cherries and on day 0, 5, and 10 for strawberries in triplicate using clear juice. This juice was extracted from the fruit blended at a high speed in a tissue homogenizer (Ultra-Turrax T-25, IKA Labortechnik, Saufen, Germany) and then centrifuged for 5 min at 1254× *g* (Centrifuge AVANTITM J-25, Beckman Instruments, Fullerton, CA, USA). Total soluble solids were determined with a digital refractometer (Atago model PR-32, Milano, Italy). Titratable acidity was measured from the clear juice of each sample (10 mL juice diluted in 10 mL of distilled water), and titrated automatically with 0.1 N NaOH to an 8.1 pH by using Titralab AT1000 Series (Radiometer Analytical, Villeurbanne, France) and then expressed as milliequivalents per liter (MEQ/L).

The analysis of Vitamin C followed Sanchez-Moreno et al. [19], with 10 g of each sample combined with 10 mL of a water-diluted methanol extraction solvent (ratio of 5:95 v/v). The sample was homogenized with an Ultra-Turrax T-25 Tissue homogenizer (IKA Labortechnik, Saufen, Germany) for 1 min at a relative centrifugal force of 45,158× *g*, and then centrifuged for 15 min at 1254 *g* (Centrifuge AVANTITM J-25, Beckman Instruments, Fullerton, CA, USA). The pH of the samples was adjusted to 2.2–2.4, and the extract was adsorbed on a C18 Sep-Pak cartridge (Waters Associates, Milford, MA, USA). The resultant solution was added to 1,2-phenylenediamine dihydrochloride (Fluka Chemika, Neu-Ulm, Switzerland) and left for 37 min in the dark at room temperature before HPLC analysis. The chromatographic system (Agilent Technologies, Santa Clara, CA, USA) was equipped with a Kinetex-C18 column (4.6 × 150 mm, 5 μm, Phenomenex, Torrance, CA, USA), a pump, and a diode array detector. The mobile phase (isocratic) consisted of 0.05 mol·L^−1^ of monobasic potassium phosphate and 0.005 mol·L^−1^ of cetrimide in a water-diluted methanol solution (5:95 v/v). The flow rate was 0.9 mL min^−1^, and the total run time was 15 min. The detector was set at 261 nm for ascorbic acid and 348 nm for dehydroascorbic acid. Vitamin C content was calculated as the sum of ascorbic and dehydroascorbic acid content and expressed as mg 100 g^−1^ of fresh weight.

All standards and reagents were of analytical purity “pro-analysis” and purchased from SIGMA (Sigma Italiana SRL, Ozzano Emilia, Italy).

### 2.6. Disease Incidence

The fruits were examined for signs of fungal infection (mold) at 5, 10, and 15 days for cherries and at 5 and 10 days for strawberries [20]. Fruits with visible mold growth were considered to be decayed and were weighted. The weight of the decayed fruit was divided by the initial weight of the sample and expressed as a percentage, according to Equation (2):

DI (%) = 100 × (DW/SW)(3)

where DI is the percentage of decay incidence, SW is the sample weight, and DW is the infected fruit weight.

### 2.7. Sensory Evaluation (Cherries)

A panel of 15 judges with sensory evaluation experience assessed the changes in the sensory properties of the cherries during storage. Samples were labeled with random three-digit codes and presented to the panelists in a random order. The evaluators were asked to use an anchored line scale to rate differences between the treatments according to berry color, stem color, surface shrivel, berry decay, sweetness, acidity, juiciness, and overall quality. Cherry quality was scored along a range of 0–9, with “low intensity” and “high intensity” being the anchor points of the scale (0 = poor/no quality, 4.5 = medium quality, 9 = excellent characteristics) [21,22]. All eight of the parameters were evaluated after 5, 10, and 15 days.

### 2.8. Visual Quality Evaluation (Strawberries)

The visual quality evaluation of the strawberries was performed every day during the 10 days of storage by 15 trained judges that assigned a score (1–5) to the sample: 1 corresponded to very poor quality, 3 to medium/acceptable quality, and 5 to excellent quality [23]. The assessors were trained to detect minimal changes in fruit appearance and had extensive experience using the scale.

### 2.9. Statistical Analysis

The experiments were carried out using a completely randomized design with three replicates. All data parameters were expressed as the mean ± SD. One-way analysis of variance (ANOVA) was applied between the Life^+^ and control fruit for each storage time using Statistica software (Statistica 10.0, Statsoft Inc., Tulsa, OK, USA). The sources of variation were the packaging treatment and storage time. Means were compared using Tukey’s test (*p* ≤ 0.05).

## 3. Results and Discussion

### 3.1. Headspace Gas Composition

Packaging cherries with a Topaz film creates a modified internal atmosphere (reduced O_2_ and increased CO_2_ content) via the metabolism of the fruit. The Life^+^ packaged cherries reached a steady-state atmosphere (14 kPa for O_2_ and 8 kPa for CO_2_) after just one day. The same trend was reported in a previous study by Caner et al. (2008), in which the observed steady-state O_2_ concentration was above that recommended for O_2_ (3–10 kPa) for MAP cherries [24]. In the case of cherries, the observed CO_2_ levels fell within the recommended levels (8–10 kPa) in MAP storage conditions [25], a result that is attributed to the ability of the film to regulate O_2_ and CO_2_ concentrations inside the packaging. By comparison, no O_2_ or CO_2_ changes were detected in the control packages due to the presence of holes on the container.

The O_2_ concentrations inside all the Life^+^ packaged strawberries decreased rapidly during the initial days of storage, and a steady-state concentration was established between the second and third day. According to Chen et al. [26], MAP packaging takes approximately three days to generate a steady-state atmosphere passively through produce respiration. In this study, the O_2_ concentration fell from 21 kPa to a steady-state value of about 12 kPa, whereas the CO_2_ concentration hovered around 10 kPa in both treatments following an initial rapid increase in CO_2_ concentration from 0 kPa to 6 kPa inside all the packages during the first day. Overall, the study values were similar to previous strawberry research [27,28]. As expected, the O_2_ and CO_2_ values were unchanged in the clamshell packages due to the presence of holes on the containers.

### 3.2. Weight Loss

The weight lost from the loss of water in the cherries increased during storage in both treatments (Table 1). In general, losses were significantly smaller (below 0.12%) after 15 days of storage with the use of the innovative packaging. The decreasing water losses during storage effectively limited undesirable wilting, shriveling, and color changes. Moreover, it is assumed that the Life^+^ system maintained a higher relative humidity in the packs due to the heat sealing that reduced the vapor pressure deficit and, consequently, lowered the transpiration.

The weight loss of the strawberry samples in storage under passive EMAP and the control is shown in Table 2. When fruits like strawberries are stored in MAP, their weight loss is usually very low (0.2%–2%) [27,28,29,30]. Life^+^ creates a modified atmosphere with higher CO_2_ and lower O_2_ concentrations around the produce, which slows metabolic processes and transpiration. Moreover, due to an adequate water vapor barrier inside the packaging, the samples yielded an extremely small weight loss compared to the control. By the end of the storage, the cumulative weight losses in the Life^+^ samples averaged <0.1%, compared to an average of 3.5% for the control fruit.

### 3.3. Quality Parameter

At harvest, the chroma index (color intensity or saturation) for cherries was 17.71, which diminished significantly during storage in both treatments. Despite the trend, the Life^+^ packaged cherries had significantly higher values throughout storage (Table 3), which likely indicates that the fruits stored with EMAP retained their vivid color longer during storage. Fruit lightness (or brightness), which measures the ratio of white (100) to black (0) color, decreased significantly during storage. On the other hand, the L * value differences among the treatments were not significant, with the exception of Life^+^ fruits after 10 days of storage. In this case, the cherries had a more brilliant and lighter color. In all cases, the study results aligned with those of Serrano et al. and Valero et al. [31,32].

The total soluble solid (TSS) content is related to the taste for a variety of fruit, including strawberries and cherries, and is commonly used to establish the optimal harvest and postharvest quality. The initial TSS (17.5%) of fresh cherries indicated good maturity, as described by Kappel et al. [33]. In this study, the TSS values for the Life^+^ samples initially decreased and then increased slightly during storage as a result of ripening (Table 4). The control cherry TSS values did not change during storage.

Storage time caused a significant decrease in the acidity of cherry samples (Table 4). Among treatments, TA was significantly higher with the Life^+^ system than with the control. This effect is considered important because the preservation of TA during storage maintains the aroma and taste of freshly-harvested cherries. A decrease in TA is a natural trend during fruit ripening. However, higher values found in active packaging result in higher quality during storage. Caner et al. [10] reported the same results in a study where the equilibrium of the atmosphere reached in the packaging affected the senescence and significantly reduced the loss of quality.

The pH level increased slightly during storage under both treatments (Table 4). At the end of storage, significant differences were detected between the EMAP and the control, which is similar to other results [34,35,36]. The equilibrium-modified-atmosphere inside the Life+ packaging had no significant effect on pH.

The Vitamin C content in the cherry samples was measured on day 0 and day 15 of cold storage. Significant differences were observed between the control and treatment (Table 4). Vitamin C content losses in the control were significantly higher than those in the Life^+^ samples. Vitamin C declines rapidly after harvest and during storage. In general, MAP-treated cherries maintained their initial level of Vitamin C content during storage by establishing an atmosphere (appropriate CO_2_ and low O_2_ concentrations) slowed fruit respiration and decreased senescence. Qian et al. [37] described how an appropriate concentration of CO_2_ prevents declines in vitamin C due to the reduction of enzymatic synthesis.

The lightness (L * value) and intensity of color (C * value) of “Marmolada” strawberries are displayed in Table 5. The chroma index (C *) reflects color saturation; higher values correspond to the development of an intense red color. In this case, the higher values of C * measured on day 5 and 10 in the Life^+^-packaged fruit indicated that the red color of the strawberries was purer and more vivid than that of the control.

The L * values significantly decreased during storage as a consequence of the pink-red color that naturally develops when strawberries ripen. In this study, the L * value of the actively-packaged fruit remained higher than that of the control packages. The same trend was observed for ‘Selva’ strawberries during another postharvest study [38].

The results from the evaluation of strawberry quality during storage are summarized in Table 6. In this study, the TSS of the ‘Marmolada’ strawberries in EMAP was relatively stable throughout storage, whereas TSS declined significantly in the control samples. The lower values in the control can be explained by the use of sugars for respiration during storage [36].

As with TSS, the total acid content (TA) of the control strawberries decreased significantly during storage (Table 6), probably due to greater respiration and higher amounts of organic acids that were used as substrates in this process. Specifically, a comparison of the TA postharvest quality results of the control-packaged fruit to the Life^+^ system-packaged fruit showed sizeable decreases in the former and only a slight decrease in the latter packaging over time.

Measurements of pH in both the control and Life^+^ fruit were close to 4.5. No significant variation in pH was detected during storage for either treatment (Table 6). Previous studies [39,40], have also found the pH to be unaffected by storage or MAP packaging.

### 3.4. Decay Incidence

The effect of packaging on cherry decay is shown in Table 7. Fruit was considered unacceptable when one disorder, mainly fungal spoilage, was visible, according to the parameters established by Sanz et al. [41]. Disorders were evident on the fifth day of storage. The number of infected fruit grew significantly with storage for both treatments. The percentage of decay was lower in the Life^+^ samples than in the control, and a difference was significant at every time of measurement. After 15 days, the control exhibited the greatest level of visible disorders with about 5% of the cherries having infections (Table 7). These data support the Life^+^ system claims that their system protects fruit against fungal and microbial attack, and that this effect persists throughout storage, with a positive and effective impact on waste reduction at the retail and consumer levels.

This study demonstrated that the decay incidence in strawberries was significantly affected by treatment. Five percent of the control berries showed fungal development after five days, and this percentage grew during storage. On the other hand, the Life^+^ system fruit’s decay was delayed and significantly less significant. Five days after storage, no disorders were observed. By the end of storage, the number of infected strawberries in the active packaging reached 8%, compared with 30% in the control. The effect of the pad on decay reduction was clearly observed in the active packaging.

### 3.5. Sensory Analysis (Sweet Cherries)

**Figure 1:**
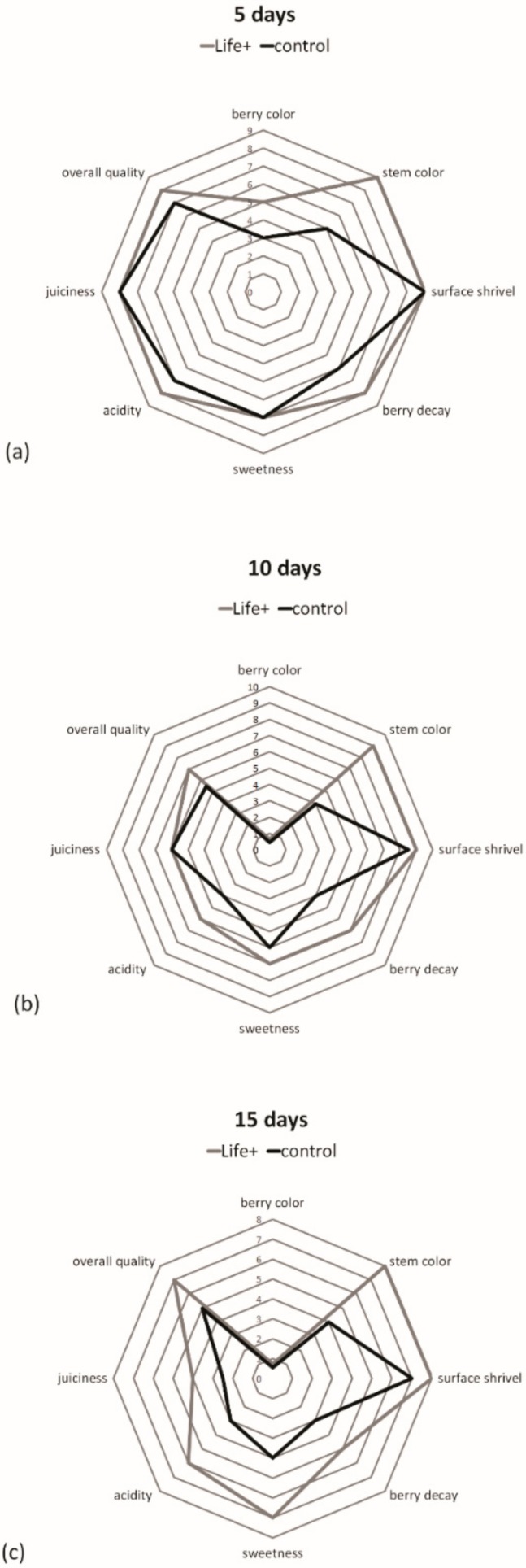
Sensory evaluation of sweet cherries after 5 (**a**), 10 (**b**), and 15 (**c**) days of storage at 6 °C.

The sensory quality of the cherries decreased during storage in both treatments. After five days, cherries stored in active packaging had better quality in terms of stem color, decay, acidity, and overall quality compared to the control. During storage, evaluators assigned lower scores for stem color and berry decay (quality attributes) to the control than to the EMAP samples, akin to the ratings in Serrano et al. [31]. At the end of storage, the control had a greater loss in quality than the Life^+^ packaged samples. Moreover, the overall quality, acidity, and sweetness indicated that the EMAP-stored cherries had better sensory quality than those stored in traditional containers (Figure 1).

### 3.6. Visual Quality (Strawberries)

The overall visual quality score of strawberries decreased gradually during storage in both treatments (Table 8). The EMAP samples maintained excellent quality (score 5) for six days, after which their quality declined. The control samples received lower and non-marketable scores after eight days. Decay was the main issue. The high susceptibility of strawberries to *Botrytis cinerea* shortens the shelf-life of strawberries [42]. Here, approximately 30% of fruit was infected after 10 days.

## 4. Conclusions

Maintaining the quality of highly-perishable fruit, such as sweet cherries and strawberries, is challenging. This study demonstrated that EMAP packaging sustained an atmosphere of higher CO_2_ and lower O_2_ concentrations, which reduced decay rates, maintained higher acidity and Vitamin C content, and preserved the bright red color of the fruits during a storage time of 5 days for strawberries and 15 days for sweet cherries. The Life^+^ system reduced weight loss and maintained sensory quality in cherries. The use of the antimicrobial pad, a component of the Life^+^ system, demonstrated a capability to reduce microorganism growth, which, in turn, has the potential to reduce postharvest waste. In conclusion, EMAP equipped with an antimicrobial pad can be used to store sweet cherries and strawberries. This innovative packaging offers a viable alternative for producers that need prolonged storage. One example that is especially enticing for the application of the Life^+^ system is its use in combination with refrigerated storage in situations of longer pre-distribution transportation times. This combination represents expanded market opportunities for producers.

## Figures and Tables

**Table 1:** Weight loss of sweet cherries stored at 6 °C for 15 days.

	Treatment	Days of Storage		
		5	10	15
Weight loss (%)	Life^+^	0.05 ± 0.01 ^b^^,C^	0.08 ± 0.01 ^b,B^	0.12 ± 0.03 ^b,A^
	Control	0.95 ± 0.25 ^a,B^	1.75 ± 0.33 ^a,A,B^	2.32 ± 0.41 ^a,A,1^

^1^ Means values ± standard deviation followed by the same letter are not significantly different at a *p* ≤ 0.05 level. Lowercase letters in the same column are used to compare treatments. Uppercase letters in the same row are used to compare storage times.

**Table 2:** Weight loss of strawberries stored at 6 °C for 15 days.

	Treatment	Days of Storage	
		5	10
Weight loss (%)	Life^+^	0.05 ± 0.01 ^b,B^	0.09 ± 0.02 ^b,A^
	Control	1.74 ± 0.35 ^a,B^	3.58 ± 0.84 ^a,A,1^

^1^ Mean values ± standard deviation followed by the same letter are not significantly different at *p*≤ 0.05 level. Lowercase letters in the same column are used to compare treatments. Uppercase letters in the same row are used to compare storage times.

**Table 3:** Color parameters of sweet cherries stored at 6 °C for 15 days.

	Treatment	Day of Storage			
		0	5	10	15
C *	Life^+^	17.7 ± 1.1 ^a,A^	13.4 ± 0.9 ^a,B^	15.5 ± 1.2 ^a,B^	15.4 ± 0.8 ^a,B^
	Control	17.7 ± 1.1 ^a,A^	9.8 ± 1.3 ^b,B^	12.6 ± 1.4 ^b,B^	12.1 ± 0.7 ^b,B^
L *	Life^+^	25.2 ± 1.7 ^a,A^	20.5 ± 1.1 ^a,B^	19.2 ± 1.1 ^a,B^	15.6 ± 1.1 ^a,B^
	Control	25.2 ± 1.7 ^a,A^	23.2 ± 1.2 ^a,B^	16.2 ± 0.9 ^b,B^	15.2 ± 0.9 ^a,B,1^

^1^ Mean values ± standard deviation followed by the same letter are not significantly different at the *p* ≤ 0.05 level. Lowercase letters in the same column are used to compare treatments. Uppercase letters in the same row are used to compare storage times.

**Table 4:** The evolution of the quality parameters of sweet cherries (Total Soluble Solids (TSS), Titratable Acidity (TA), and Vitamin C content) stored at 6 °C for 0 to 15 days.

	Treatment	Day of Storage			
		0	5	10	15
TSS (° Brix)	Life^+^	17.5 ± 0.9 ^a,A^	16.4 ± 0.9 ^a,B^	17.5 ± 0.7 ^a,A^	17.4 ± 1.1 ^a,A^
	Control	17.5 ± 0.9 ^a,A^	17.6 ± 1.0 ^a,B^	17.7 ± 0.9 ^a,A^	17.3 ± 2.0 ^a,A^
TA (meq/L)	Life^+^	89.9 ± 2.2 ^a,A^	71.2 ± 2.3 ^a,B^	82.5 ± 1.1 ^a,A^	70.4 ± 2.3 ^a,B^
	Control	89.9 ± 2.2 ^a,A^	69.7 ± 0.9 ^a,b,B^	67.8 ± 1.7 ^b,B^	65.0 ± 2.4 ^b,B^
pH	Life^+^	4.0 ± 0.3 ^a,B^	4.1 ± 0.1 ^b,B^	4.2 ± 0.1 ^a,A^	4.3 ± 0.1 ^b,A^
	Control	4.0 ± 0.3 ^a,B^	4.2 ± 0.1 ^a,A^	4.2 ± 0.2 ^a,A^	4.3 ± 0.1 ^a,A^
Vitamin C (mg * 100 g^−1^ FW)	Life^+^	9.8 ± 1.1 ^a,A^			9.7 ± 3.2 ^a,A^
	Control	9.8 ± 1.1 ^a,A^			6.1 ± 2.1 ^b,B,1^

^1^ Mean values ± standard deviation followed by the same letter are not significantly different at *p* ≤ 0.05 level. Lowercase letters in the same column are used to compare treatments. Uppercase letters in the same row are used to compare storage times.

**Table 5:** Color parameters of strawberries stored at 6 °C for 10 days.

	Treatment	Day of Storage		
		0	5	10
C *	Life^+^	31.1 ± 3.1 ^a,B^	36.4 ± 2.1 ^a,A,B^	39.2 ± 3.2 ^a,A^
	Control	31.1 ± 3.1 ^a,A^	30.3 ± 1.8 ^b,A^	13.6 ± 0.9 ^b,B^
L *	Life^+^	37.4 ± 2.0 ^a,A^	34.3 ± 2.2 ^a,B^	31.7 ± 3.2 ^a,C^
	Control	37.4 ± 2.0 ^a,A^	32.9 ± 1.9 ^b,B^	29.1 ± 3.4 ^b,C,1^

^1^ Mean values ± standard deviation followed by the same letter are not significantly different at a *p* ≤ 0.05 level. Lowercase letters in the same column are used to compare treatments. Uppercase letters in the same row are used to compare storage times.

**Table 6:** Evolution of quality parameters for strawberries (Total Soluble Solids (TSS), Titratable Acidity (TA), and Vitamin C content) during cold storage at 6 °C for 0 to 10 days.

	Treatment	Day of Storage		
		0	5	10
TSS (° Brix)	Life^+^	7.0 ± 0.3 ^a,A^	6.6 ± 0.5 ^a,A^	6.9 ± 0.3 ^a,A^
	control	7.0 ± 0.3 ^a,A^	5.7 ± 0.2 ^b,B^	5.9 ± 0.4 ^b,B^
TA (meq/L)	Life^+^	106.7 ± 5.2 ^a,A^	105.2 ± 4.8 ^a,A^	104.1 ± 3.2 ^a,A^
	control	106.7 ± 5.2 ^a,A^	84.6 ± 3.9 ^b,B^	78.3 ± 6.8 ^b,B^
pH	Life^+^	3.4 ± 0.4 ^a,A^	3.5 ± 0.4 ^a,A^	3.5 ± 0.3 ^a,A^
	control	3.4 ± 0.4 ^a,A^	3.7 ± 0.34 ^a,A^	3.6 ± 0.4 ^a,A^
Vitamin C (mg * 100 g^−1^ FW)	Life^+^	52.8 ± 3.0 ^a,A^	46.1 ± 2.1 ^a,B^	46.2 ± 3.0 ^a,B^
	control	52.8 ± 3.0 ^a,A^	41.7 ± 2.0 ^b,B^	41.8 ± 1.2 ^b,B,1^

^1^ Mean values ± standard deviation followed by the same letter are not significantly different at a *p* ≤ 0.05 level. Lowercase letters in the same column are used to compare treatments. Uppercase letters in the same row are used to compare storage times.

**Table 7:** Decay incidence (%) in sweet cherries stored at 6 °C after 5 to 15 days.

	Treatment	Day of Storage		
		5	10	15
Decay incidence (%)	Life^+^	0.21 ± 0.05 ^b,B^	0.45 ± 0.09 ^b,B^	0.62 ± 0.03 ^b,A^
	Control	1.00 ± 0.12 ^a,C^	2.02 ± 0.19 ^a,B^	4.78 ± 0.85 ^a,A,1^

^1^ Mean values ± standard deviation followed by the same letter are not significantly different at a *p* ≤ 0.05 level. Lowercase letters in the same column are used to compare treatments. Uppercase letters in the same row are used to compare storage times.

**Table 8:** Visual quality evaluation of strawberries stored at 6 °C for 10 days.

Treatment	Day of Storage								
	0	1	2	3	6	7	8	9	10
Life^+^	5.0 ± 0.0	5.0 ± 0.0	5.0 ± 0.0	5.0 ± 0.0	5.0 ± 0.0	4.5 ± 0.4	3.5 ± 0.4	3.0 ± 0.6	2.5 ± 0.7
Control	5.0 ± 0.0	5.0 ± 0.0	4.5 ± 0.3	4.5 ± 0.4	3.5 ± 0.5	3.0 ± 0.6	2.5 ± 0.5	1.5 ± 0.4	1.0 ± 0.0 ^1^

^1^ Means values ± standard deviation.
